# Hemifacial microsomia associated with vascular malformation of vertebral: A case report

**DOI:** 10.1016/j.ijscr.2023.108906

**Published:** 2023-10-03

**Authors:** Arif Tri Prasetyo, Indri Lakhsmi Putri, Anggun Esti Wardani

**Affiliations:** a Division of Plastic Reconstructive and Aesthetic Surgery, Department of Surgery, Faculty of Medicine, Universitas Padjadjaran, Bandung, Indonesia; b Department of Plastic Reconstructive and Aesthetic Surgery, Faculty of Medicine, Universitas Airlangga, Surabaya, Indonesia; c Department of Radiology, Faculty of Medicine, Universitas Airlangga, Surabaya, Indonesia; d Universitas Airlangga Hospital, Surabaya, Indonesia; e Airlangga Health Science Institute, Indonesia

**Keywords:** Hemifacial microsomia, Vertebral vein malformation, Microtia, CT angiography, Vascular anomaly

## Abstract

**Introduction and importance:**

Hemifacial microsomia (HFM) is a complex congenital facial anomaly characterized by a wide spectrum of clinical features, which encompass the facial skeleton and other organ systems. Currently, there is no evidence to suggest an association between Hemifacial Microsomia and vascular malformations, whether of the vertebral or any other kind.

**Case presentation:**

Reporting a case of a 12-year-old male diagnosed with Hemifacial Microsomia (HFM) and left Microtia. The patient had previously undergone left auricle reconstruction; however, unfortunately, the flap resulted in necrosis. In our next step, we intend to proceed with further reconstruction. Before this, we plan to perform CT angiography to identify viable flap options for effectively closing the auricular defect. During this evaluation, we identified an anomaly structure in the vertebral vascularization.

**Clinical discussion:**

During the CT angiography, we found a vascular malformation in the vertebral region. This anomaly manifested as tortuosity in the left vertebral vein, with the diameter on the left side being larger than that on the right. Additionally, the diameter of the left internal jugular artery was found to be smaller than its counterpart on the right. The maxillary artery of the left side was larger than the right. Notably, there was an absence of a submental artery on the left side, and a hypoplasia of the left angularis artery was observed.

**Conclusion:**

Hemifacial microsomia could be associated with other malformations. Despite the fact that vertebral artery anomaly is not considered common anomaly in HFM, it is mandatory to perform CT angiography before reconstructive surgery, considering the possibility of massive bleeding during the operation.

## Highlights

• Hemifacial microsomia (HFM) consist of complex facial malformation with a wide spectrum of clinical features involving the facial skeleton and other organ systems
• One major concern related to flap technique in HFM reconstruction is anatomy of vessels in head and neck region
• HFM with some anomaly vascular which make the reconstruction become challenging.

## 1 Introduction

Hemifacial microsomia (HFM) is a variable, complex congenital anomaly that is most strictly defined as asymmetric hypoplasia of the face and ear, a large host of genetic and teratogenic associations, and a wide spectrum of clinical features involving the facial skeleton and other organ systems [1,2]. HFM is the second most common congenital anomaly of the head and neck region, with an incidence as high as 1 in 3500 live births. There is a wide variety of pathologic expression of craniofacial microsomia in the following anatomic regions: jaws, other craniofacial skeletal components, muscles of mastication, ears, soft tissue, and nervous system Major manifestations of HFM are orbital distortion, mandibular hypoplasia, ear anomalies, nerve involvement, and soft tissue deficiency (OMENS classification) [3]. Hearing loss, mastication impairment, breathing problems, speech impediments, and sleep disorders can occur as part of HFM. To improve function and appearance, a range of corrective surgeries may be indicated. Treatments and procedures can occur over many years and undoubtedly are disruptive to both child and family. HFM may have long-term effects on psychological development and social well-being, due to unusual facial appearance, functional problems, and medical treatments [4].

The etiology of HFM is unclear: teratogenic agents, socioeconomic risk factors, and chromosomal abnormalities have been described. Genetic transmission is suspected because several families have been described with an autosomal-dominant pattern if inheritance [5]. The reconstruction of HFM is challenging. The modality of flap technique depends on the patient anatomy.

One major concern related to flap technique in HFM reconstruction is anatomy of vessels in head and neck region. The presence of a vertebral variant must be considered due to difficulties in detection. Some patients report some symptoms such as dizziness and vertigo. Another importance seeking any possible anomaly is alteration in hemodynamics and intracerebral malformation [6].

Arteriovenous malformation of the vertebral artery in childhood is a rare condition. We reported a HFM with some anomaly vascular which make the reconstruction become challenging.

## 2 Presentation of case

**Fig. 1:**
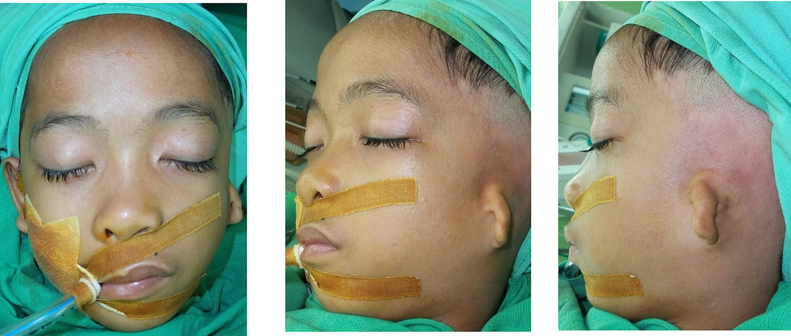
Hemifacial microsomia with microtia of left ear before first surgery.

The case is about vertebral artery malformation on HFM patient. As shown in Fig. 1, a 12-year-old male was admitted to the hospital for the preparation of left auricle reconstruction. Prior to this admission, the first stage reconstruction of the left auricle had already been completed. As per the initial plan, this surgery aimed to elevate the left auricle cartilage, harvest the cartilage, and place it on the left retro auricle. The bone graft would then be covered with a temporoparietal fascia flap, followed by a full-thickness skin graft. Unfortunately, the flap from the second stage procedure resulted in failure.

**Fig. 2:**
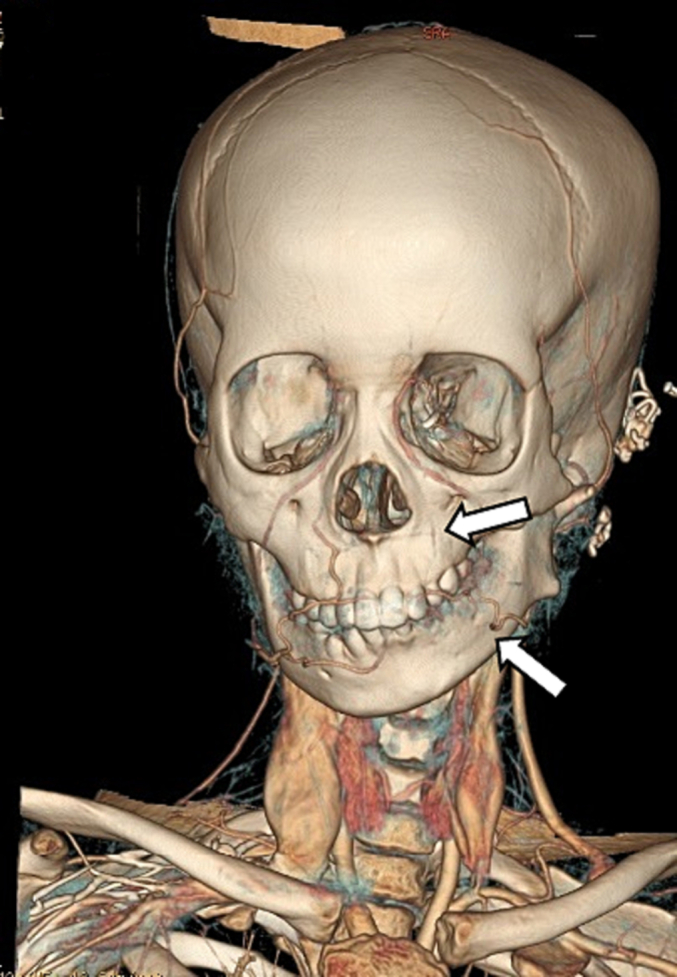
From the CT-Angiography there is agenesis of left submental artery and hypoplasia of left angularis artery.

**Fig. 3:**
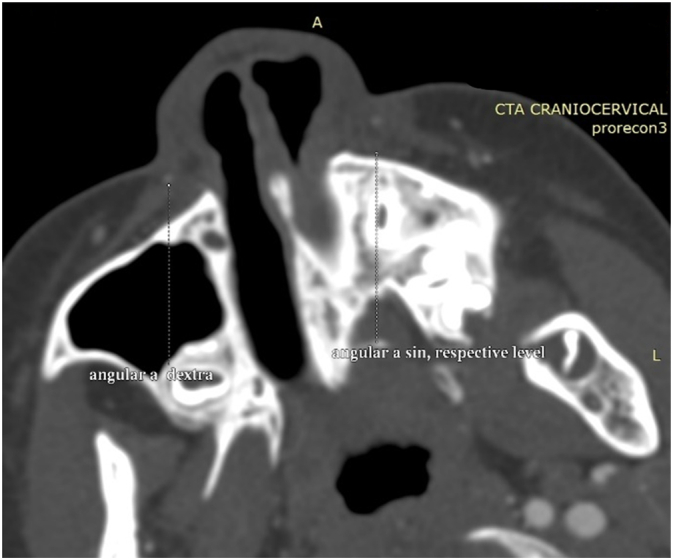
Comparation between right angularis artery and respective level of left angularis artery.

**Fig. 4:**
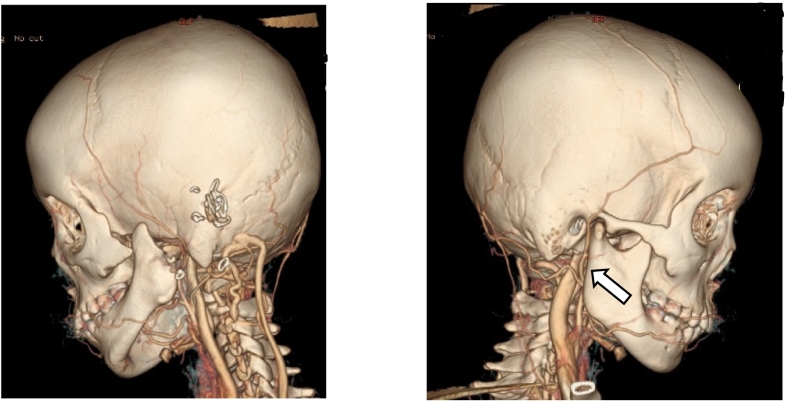
From the CT-Angiography there is a tortuosity of left vertebral vein with the diameter of the left is bigger than the right.

**Fig. 5:**
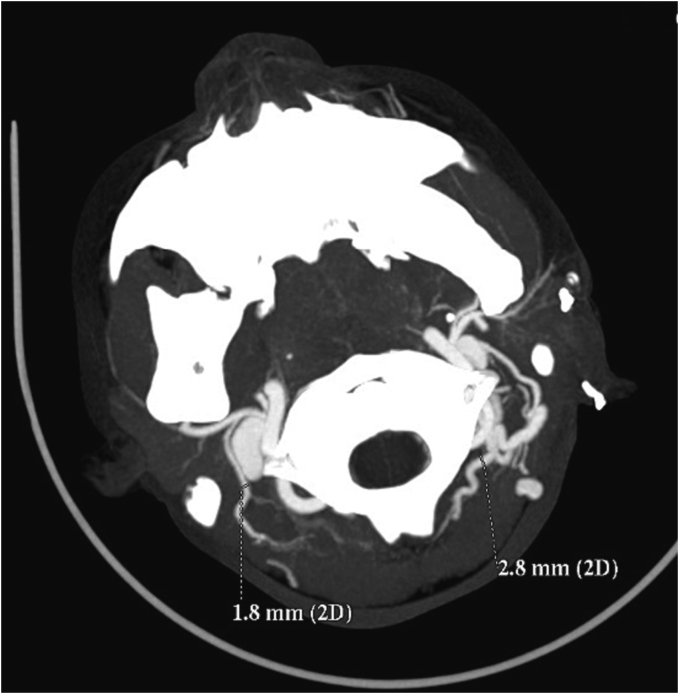
From the CT-Angiography there is different size of internal jugular artery on the right and left side.

**Fig. 6:**
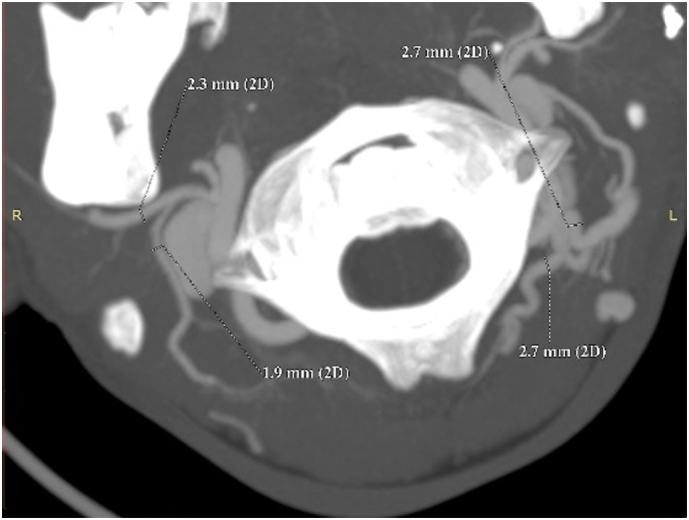
The diameter of maxillary artery is different both side. The right side is 2.3 mm and the left side is 2.7 mm.

We planned to use a deep temporal fascia pedicled flap to cover the cartilage defect. To ensure accuracy, we conducted a CT Angiography to determine the presence of the deep temporal artery. In Figs. 2 and 3, an angiography was performed to locate a suitable vessel for pedicle attachment in the upcoming reconstruction. It was discovered that the patient had an anatomical malformation of the vertebral artery during the angiography. Fig. 4 reveals tortuosity in the left vertebral vein, with the diameter being larger on the left side compared to the right. On Fig. 5, it can be observed that the internal jugular artery on the right side is smaller than on the left side, with respective diameters of 1.8 mm and 2.8 mm. Fig. 6 illustrates the discrepancy in diameter of the maxillary artery between both sides, with the right side measuring 2.3 mm and the left side 2.7 mm. There is no submental artery on the left side, and there is a hypoplasia of the angularis artery on the left side as well. The work has been reported in line with the SCARE criteria [7].

## 3 Discussion

Hemifacial microsomia (HFM) is primarily a congenital anomaly of first and second branchial arches [8,4] and associated with certain malformations. An alternative and more plausible hypothesis is that HFM results from defective neural crest cell (NCC) development [9]. Any anomaly in HFM can be explained by tracing it to these facts.

A series of case reports indicated some significant malformations in the OMENS classification system and extracranial anomalies. In this study, each patient was categorized using OMENS and examined for any additional anomalies in the central nervous system, cardiovascular system, pulmonary system, renal system, gastrointestinal system, and skeletal system. However, this study did not mention any vascular malformations [10].

It is crucial to comprehend the nature of HFM. One possible syndromic explanation may be Goldenhar Syndrome (GS), also known as oculo-auricular-vertebral dysplasia. GS is a rare condition characterized by malformations of the eyes and ears. It represents a developmental abnormality involving the first and second branchial arches as well as vertebral bodies, leading to the triad of craniofacial microsomia, along with ocular and vertebral abnormalities [11]. A case report on Goldenhar Syndrome (GS) may shed light on vascular anomalies, as it has been noted to be one of the associated findings in some cases.

Microtia has become one of the concerns in cases of hemifacial microsomia. The initial stage of microtia reconstruction involves placing a cartilage network into a skin pocket. In the subsequent stage of reconstruction, a flap around the scalp is required to cover the cartilage. The significance of CT angiography in cases of hemifacial microsomia with microtia lies in determining the appropriate flap for cartilage coverage. Selecting the correct pedicle for the flap is crucial for its success. Based on our case, we believe that CT angiography is essential not only for selecting the appropriate pedicle flap but also for ensuring the safety of the surgery.

In this case, the patient has a larger diameter of the maxillary artery on the left side. Since we know that the superficial and deep temporal arteries are branches of the maxillary artery, we believe that the increased diameter of the artery may lead to a higher probability of bleeding intraoperatively.

## 4 Conclusion

While vertebral artery anomalies are not commonly associated with HFM, it is crucial to do angiography of major vessels in the head and neck region prior to undertaking significant corrective surgery.

## Consent

Written informed consent was obtained from the patient's parents/legal guardian for publication and any accompanying images. A copy of the written consent is available for review by the Editor-in-Chief of this journal on request.

## Ethical approval

We have conducted an ethical approval base on Declaration of Helsinki at Ethical Committee in Universitas Airlangga Hospital, 15th September 2018.

## Funding

There was no any financial disclosure or support for this study.

## Author contribution

Arif Tri Prasetyo: Study concept or design, data collection, data analysis, interpretation, writing the paper.

Indri Lakhsmi Putri: Study concept or design, data collection, data analysis, interpretation, writing the paper.

Anggun Esti Wardani: Study concept or design, data collection, data analysis, interpretation, writing the paper.

## Guarantor

Arif Tri Prasetyo.

Indri Lakhsmi Putri.

Anggun Esti Wardani.

## Research registration number

This study does not require registration.

## Conflict of interest statement

The authors declare no conflicts of interest related to this study.
